# Mechanism Based Flow Stress Model for Alloy 625 and Alloy 718

**DOI:** 10.3390/ma13245620

**Published:** 2020-12-09

**Authors:** Andreas Malmelöv, Martin Fisk, Andreas Lundbäck, Lars-Erik Lindgren

**Affiliations:** 1 Division of Mechanics of Solid Materials, Luleå University of Technology, SE-971 87 Luleå, Sweden; andreas.lundback@ltu.se (A.L.); lars-erik.lindgren@ltu.se (L.-E.L.); 2 Department of Materials Science and Applied Mathematics, Malmö University, SE-205 06 Malmö, Sweden; martin.fisk@mau.se; 3 Division of Solid Mechanics, Lund University, P.O. Box 118, SE-221 00 Lund, Sweden

**Keywords:** material model, flow stress model, dislocation density, Inconel, stress relaxation

## Abstract

To predict the final geometry in thermo-mechanical processes, the use of modeling tools is of great importance. One important part of the modeling process is to describe the response correctly. A previously published mechanism-based flow stress model has been further developed and adapted for the nickel-based superalloys, alloy 625, and alloy 718. The updates include the implementation of a solid solution strengthening model and a model for high temperature plasticity. This type of material model is appropriate in simulations of manufacturing processes where the material undergoes large variations in strain rates and temperatures. The model also inherently captures stress relaxation. The flow stress model has been calibrated using compression strain rate data ranging from 0.01 to 1 s^−1^ with a temperature span from room temperature up to near the melting temperature. Deformation mechanism maps are also constructed which shows when the different mechanisms are dominating. After the model has been calibrated, it is validated using stress relaxation tests. From the parameter optimization, it is seen that many of the parameters are very similar for alloy 625 and alloy 718, although it is two different materials. The modeled and measured stress relaxation are in good agreement.

## 1. Introduction

Alloy 625 and alloy 718 are two commonly used nickel-based superalloys. Alloy 625 is used in the aerospace, marine, petrochemical and nuclear industries owing to its high tensile, rupture and creep strength, and excellent corrosion resistance [1]. Alloy 718 is mostly used in power plants and gas turbines and is also the main superalloy utilized in jet engines due to its high-temperature strength [2]. The main difference between alloy 625 and alloy 718 is that alloy 625 is a solid solution hardened material, strengthened by Cr, Mo, Nb and Fe [3], and alloy 718 is a precipitation hardened material, mainly strengthened by the body-centered tetragonal (bct) $\gamma^{\prime \prime }$-precipitates that comprises Ni and Nb (Ni_3_Nb) [2]. Precipitation strengthening is however not considered in this work, since alloy 718 is used in its annealed state. The alloy composition is similar, although the amount of niobium content is higher in alloy 718 (4.75–5.50 wt %) compared to alloy 625 (3.15–4.15 wt %), and alloy 718 has a much higher fraction of iron, see Table 1. Both alloy 625 and 718 are commonly used in manufacturing processes such as machining, welding and additive manufacturing (AM) [4,5]. During welding and particularly AM, the material undergoes temperature fluctuations that reach several hundreds of degrees and rapidly drop close to room temperature within an interval of a few seconds. To further develop parts produced by AM, there is an interest in predicting residual strains and stresses as well as the final geometry. One of the critical aspects of modeling AM is how to treat the stress-strain material response from room temperature and up to temperatures close to the melting temperature. Machining also requires a material model that accurately models the stress–strain behavior [6]. In machining, the material undergoes severe plastic deformations at strain rates ranging from six orders of magnitude and temperatures in a wide temperature range. Roll forming and sheet metal forming are other examples where this kind of material model is useful for capturing the mechanical behavior over varying conditions.

There are two classes of material yielding models: empirically based material models that are based on mathematical expressions that reproduce the stress-strain relation and so-called physically or mechanism-based material models that account for the different strengthening mechanisms that take place in the material. A mechanism-based model is used in the current work. This material model has, for example, been used in the work by Bergström [7] and Estrin and Mecking [8]. Frost and Ashby [9] have also provided information to the model through their development of deformation mechanisms maps. Other authors are Cheng et al. [10], who apply the model to Molybdenum. In their model, the yield stress was a sum of two terms: the long-range athermal resistance to dislocation motion and a thermally activated part usually called the short-range contribution. The model captures the differences in deformation behavior at low and high strain rates. In the same way, but here changing some of the sub-models, Lindgren et al. [11] describes the yielding behavior of the duplex steel AISI 316L. Later the model was extended [12] to account for precipitate hardening in alloy 718, but the model was only calibrated versus compression tests at 400 and 600 °C. Another example is the work by Babu et al. [13], which used the model to describe the yielding behavior of Ti-6Al-4V in the temperature range of 20–1100 °C. However, for the three last cases described, they used temperature-dependent parameters, which result in a huge amount of calibration parameters. Later, Lindgren et al. [14] improved the model for AISI 316L, making many of the calibration parameters constant that was temperature-dependent. This action resulted in a reduction of calibration parameters from 45 to 17. Similarly, Yadav et al. [15] used a mechanism-based model to describe the yielding behavior of 304 HCu stainless steel. A difference from the other works described above is that they used the mobile dislocation density as an internal state variable, in addition to the forest dislocation density. Although there are many publications describing mechanism-based material models, there is no publication of which the authors are aware that describes the yielding behavior for alloy 625 and alloy 718 from room temperature up to near melting temperature (1100 °C).

The main focus of the current work is to develop and adapt a mechanism-based flow stress model for the solid solution strengthened alloy 625 and the precipitate hardened alloy 718. The future usage for the material model is manufacturing processes such as welding, additive manufacturing, machining, forming and heat treatment. It should be emphasized that alloy 718 is calibrated and validated on wrought material that is annealed prior to testing. Thus, precipitation strengthening is not modeled. When used in processes where significant precipitation formation and growth occur, the model can be combined with a contribution for precipitation hardening as in the work by Fisk et al. [12]. The material model has been calibrated versus compression tests in temperatures, ranging from room temperature up to temperatures close to the melting temperature. An important step to ensure the structural integrity of a component after manufacturing processes, such as welding and AM, is heat treatment. A stress relaxation test at 1000 °C was therefore modeled and validated for both alloy 625 and alloy 718.

## 2. Experiments

Uniaxial axisymmetric compression tests were performed on alloy 625 in the Gleeble 3800-GTC system at Luleå University of Technology using the ISO-T tungsten carbide anvils. The test specimens were cylindrical in shape, and the state of the as-delivered material was a cold drawn rod with a diameter of 12 mm and annealed for 1 h at 980 °C. The test samples were machined into final shape for testing with a diameter of 8 mm and a length of 10 mm, which gives an aspect ratio, $D_{R}$, of 1.25. The alloy 718 material was uniaxially compressed using a Gleeble 3800-GTC system at Uleåborg University. The initial state of the alloy was cold drawn and subsequently fully annealed, i.e., heat treated at 950 °C for 1 h. The chemical composition of the two materials can be seen in Table 1.

To measure and control the temperature a thermocouple of type K was welded at the middle of the sample. For alloy 625, a C-Strain gauge of caliper type with quarts jaws was used to measure the diametral change directly on the sample during the compression. For alloy 718, the strain was measured using the displacement of the end-grips. Between the ends of the sample and the tungsten carbide anvils, a thin layer of nickel paste on each side of a 0.25 mm thick graphite foil was applied in order to reduce the friction. For both alloy 625 and alloy 718, the tests were performed at nominal strain rates of 0.01–1.0 $s^{-1}$ in the temperature range from room temperature close to the melting temperature.

**Figure 1:**
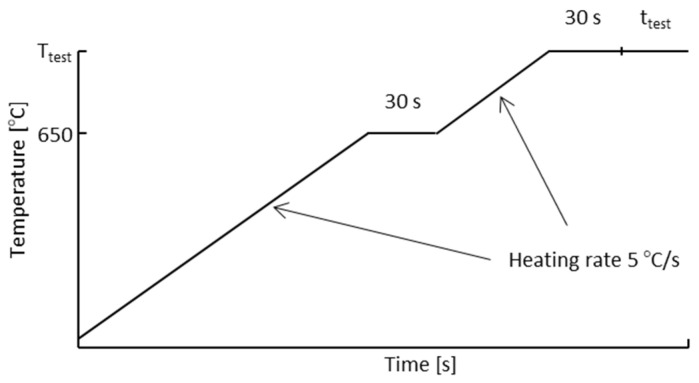
Generic temperature profile for heating of the test samples.

A generic temperature profile for the heating of the samples can be seen in Figure 1. The heating ratio for the sample is 5 °C/s. If the test is performed at a temperature above 650 °C, the sample is first left soaking at 650 °C for 30 s before it is further heated. The choice of 650 °C is due to that the current materials, alloy 625 and alloy 718, are thermally stable up to that temperature. A soaking time of 30 s is always applied before the test starts. This is to ensure a homogenous temperature within the sample.

When computing the stress and strain, it is assumed that the material is incompressible and that the test sample deforms as a perfect cylinder. That is, the volume is assumed to be constant, and we get the following relation, $L_{0}A_{0}=LA$, where L is the current length, and $L_{0}$ is the initial length of the sample. A and $A_{0}$ are the current and initial areas, respectively. The true axial strain is defined as

(1) $$\varepsilon =\ln\left(\frac{L}{L_{0}}\right)$$

Using this relation, we can express the true strain as a function of the initial diameter,$\;D_{0},$ and the current diameter, D, measured with the C-Strain gauge.

(2) $$\varepsilon_{axial}=\ln\left(\frac{A_{0}}{A}\right)=2\ln\left(\frac{D_{0}}{D}\right)$$

As the plastic strain is large compared to the elastic strain, the assumption of incompressibility should be valid.

The deviation from a perfect cylinder during the deformation is mainly caused by the friction between the sample ends and the contact platens. Particularly at high temperatures, above 900 °C, a barrel shape can be noticed on the deformed samples. The barreling coefficient, $B_{c}$, is computed as a relative measure based on the volumetric preservation according to [16]. The barreling coefficient is related to the initial length, the initial diameter, the final length, $L_{f}$, and the final diameter, $D_{f}$, according to

(3) $$B_{c}=\frac{L_{f}D_{f}^{2}}{L_{0}D_{0}^{2}}$$

The largest barrel coefficient was found for the samples tested at the highest temperature, 1150 °C. The average value of the barreling coefficient for that temperature was 1.097 which is close, but below the threshold value of 1.1 given by Roebuck et al. [16]. It is possible to compensate for the barreling on the stress–strain curves by inverse modeling in combination with a finite element analysis as shown by e.g., [17,18]. This was not done as the barreling coefficient is on the threshold value

## 3. Material Model

In alloys, many strengthening mechanisms are operative at the same time. It is common to assume that the contribution from each mechanism can be summed [6,12,13,14]. In the current work, the flow stress is formulated

(4) $$\sigma_{y}=\sigma_{G}+\sigma_{HP}+\sigma^{*}+\sigma_{s}$$

where the contributions are the following: $\sigma_{G}$ is the effect of the long-range interactions with the substructure of the immobile dislocations; $\sigma_{HP}$ is the grain boundary strengthening (Hall–Petch effect); $\sigma^{*}$ is the short-range contribution that prescribes the stress required to move dislocation past short-range obstacles, and $\sigma_{s}$ is solid solution strengthening. Other contributions to the yield strength may be added to Equation (4), such as the addition of stress required to move a dislocation though or around precipitates and solutes, or the internal friction stress to move a dislocation through a perfect lattice.

At higher temperatures, some of the mechanisms in Equation (4) are no longer dominating and replaced with new dominating mechanism(s) that control the flow stress. A relationship between flow stress and strain rate at elevated temperatures is used according to Frost and Ashby [9]. They call it power law breakdown, $\sigma_{PLB}$. This contribution is described in the end of this section. With $\sigma_{PLB}$ included, the resulting flow stress in this work is written

(5) $$\sigma_{y}=\min\left(\sigma^{*}+\sigma_{HP}+\sigma_{s},\sigma_{PLB}\right)+\sigma_{G}$$

Because $\sigma_{G}$ is an athermal contribution, it is assumed that this contribution is always active, and thereby, it is a separate term. The contributions $\sigma^{*}$, $\sigma_{HP}$ and $\sigma_{s}$ are so-called active mechanisms and control the flow stress at lower temperatures. The contribution $\sigma^{*}$ breaks down at elevated temperatures according to Frost and Ashby [9] and is replaced with a contribution for high temperature plasticity. $\sigma_{s}$ can be treated in the same way, as the contribution includes the same mechanism as $\sigma^{*}$; solutes can be seen as discrete obstacles, which the mobile dislocations must overcome. Schneibel and Heilmaier [19] also show that breakdown of $\sigma_{HP}$ occurs at elevated temperatures owing to a large variety of thermally activated mechanisms. Temperatures that are typical when $\sigma_{PLB}$ starts to dominate are above $0.6T_{m}$ and at stresses higher than $10^{-3}$ of the bulk modulus [9].

### 3.1. Long-Range Contribution

The relationship between the immobile dislocation density $\rho_{i}$ and the yield strength is usually referred as Taylors equation [20] and is given as

(6) $$\sigma_{G}=\alpha MGb\sqrt{\rho_{i}}$$

where α is a proportional factor accounting for the efficiency of dislocation density strengthening and is typically in the range of unity. M is the taylor factor that translates the effect of resolved shear stress in different slip systems into effective stress and strain quantities; G is the shear modulus that may be temperature dependent; b is Burger’s vector, and $\rho_{i}$ is the effective immobile dislocation density. In this work, we ignore that the efficiency of dislocation strengthening, α, is in a small extent dependent on the dislocation substructure, i.e., it is dependent on the current dislocation density [21]. The long-range term $\sigma_{G}$ is called an athermal stress contribution, as thermal vibration cannot assist overcoming the long-range interactions of the dislocation substructure.

The evolution in time of the immobile dislocation density, ${\dot{\rho }}_{i}$, comprises of one hardening and two recovery processes, so that

(7) $${\dot{\rho }}_{i}={\dot{\rho }}_{i}^{\left(+\right)}-{\dot{\rho }}_{i\left(glide\right)}^{\left(-\right)}-{\dot{\rho }}_{i\left(climb\right)}^{\left(-\right)}$$

where, ${\dot{\rho }}_{i}^{\left(+\right)}$ describes the hardening caused by pinning effects, and ${\dot{\rho }}_{i\left(glide\right)}^{\left(-\right)}$ and ${\dot{\rho }}_{i\left(climb\right)}^{\left(-\right)}$ describe the recovery by glide and climb, respectively. The evolution modeling of the immobile dislocation density is described in the following subsections.

#### 3.1.1. Hardening

It is assumed that the mobile dislocations move a distance before they are immobilized or annihilated. The mean distance they move is called the mean free path Λ. According to the Orowan equation (see Equation (16) below), the density of mobile dislocations and their average velocity are proportional to the plastic strain rate. In the same way, it is assumed that the rate of increase in immobile dislocations is proportional to the dislocation velocity and the mobile dislocation density and inversely proportional to the mean free path [22]. This leads to

(8) $${\dot{\rho }}_{i}^{\left(+\right)}=\frac{M}{b}\frac{1}{\Lambda }{\dot{\bar{\varepsilon \;}}}^{p}$$

The mean free path can be obtained from the fact that a dislocation moves a distance before it is hindered. Typically, this distance is proportional to the inverse sum of obstacles

(9) $$\frac{1}{\Lambda }=\left(\frac{1}{g}+\frac{1}{s}\right)$$

where g is the grain size and s is the dislocation cell or subgrain diameter. The grain size is taken as constant in the modeling, i.e., no grain growth or recrystallization is assumed. Holt [23] found out that the dislocation cell or subgrain size is proportional to $1/\sqrt{\rho_{i}}$. Equation (8) can then be expressed as

(10) $${\dot{\rho }}_{i}^{\left(+\right)}=\frac{M}{b}\left(\frac{\sqrt{\rho_{i}}}{K_{c}}+\frac{1}{g}\right){\dot{\bar{\varepsilon \;}}}^{p}$$

where $K_{c}$ is a material parameter that relate the subcell diameter to the immobile dislocation density.

#### 3.1.2. Recovery

The recovery of the material is either dynamic or static. Dynamic recovery occurs through dislocations glide, and static recovery is due to the climbing process. Recovery by dislocation glide takes place when a moving dislocation annihilates an existing immobile dislocation. Thus, the annihilation process of immobile dislocations is proportional to the mobile dislocation density, and

(11) $${\dot{\rho }}_{i\left(glide\right)}^{\left(-\right)}=\Omega \rho_{i}{\dot{\bar{\varepsilon \;}}}^{p}$$

where Ω is a recovery function that is temperature and strain rate dependent. Ω was derived by Bergström et al. [7,24] to

(12) $$\Omega =\Omega_{0}+\Omega_{r0}{\left(\frac{1}{{\dot{\varepsilon }}_{p}}\frac{D}{b^{2}}\right)}^{1/3}=\Omega_{0}+\Omega_{r0}{\left(\frac{D_{v0}}{b^{2}}\right)}^{1/3}{\left(\frac{1}{{\dot{\varepsilon }}_{p}}e^{-\frac{Q}{k_{B}T}}\right)}^{1/3}$$

where $\Omega_{0}$ and $\Omega_{r0}$ are calibration parameters, and D is a diffusivity. $k_{B}$ is Boltzmann’s constant, and T is the absolute temperature. The activation energy Q is a calibration parameter in the current work. The same approach was used in Lindgren et al. [14]. The model for dynamic recovery will not work for very low plastic strain rates since that will give an infinitely large Ω. This can be managed by introducing a maximum diffusion distance that can complete annihilation; see Lindgren et al. [14]. This distance is the product of the calibration parameter $x_{d}^{max}$ and the burgers vector.

The static recovery is due to the climbing process. Nes [25], Sandström and Lagneborg [26] indicate that this recovery should be proportional to $\rho_{i}^{2}$. The model for the static recovery, or recovery by climb, is here taken as [14]

(13) $${\dot{\rho }}_{i\left(climb\right)}^{\left(-\right)}=c_{\gamma }D_{v}\frac{Gb^{3}}{k_{B}T}\left(\rho_{i}^{2}-\rho_{i0}^{2}\right)$$

where $c_{\gamma }$ is a calibration parameter; $\rho_{i}$ is the instantaneous immobile dislocation density; $\rho_{i0}$ is the initial dislocation density, and $D_{v}$ is the temperature dependent lattice diffusion. The static recovery has a strong temperature dependency through the diffusion parameter that can be written

(14) $$D_{v}=D_{v0}e^{-\frac{Q_{v}}{k_{B}T}}$$

Some also used a model for vacancy generation and annihilation [11,13]. The fraction of vacancies is then treated as an internal state variable and included in Equation (13).

### 3.2. Hall–Petch Effect

The Hall–Petch equation describes the relationship between the grain size and the stresses that is needed to overcome yielding. During deformations, dislocations are piled-up at grain boundaries. In order to continue into the adjacent grain, the dislocation must change direction, which requires much energy. Smaller grains result in faster immobilization and thus also faster strain hardening. The contribution accounts for temperature dependency, by using the temperature dependent shear modulus, G(T), which is divided with the shear modulus at room temperature, $G_{RT}$. This can be formulated [14]

(15) $$\sigma_{HP}=k_{HP}\frac{G\left(T\right)}{G_{RT}}\frac{1}{\sqrt{g}}$$

where $k_{HP}$ is the room temperature Hall–Petch coefficient. Experimental values in the literature for the room temperature dependent Hall–Petch coefficient in nickel based superalloys are in the range $710–1060\;\mathrm{MPa}\sqrt{\mu m}$ [27,28]. In this work, a coefficient value of $750\;\mathrm{MPa}\sqrt{\mu m}$ has been used for both alloy 625 and alloy 718. In this work, we have used a grain size, g, of 20 μm.

### 3.3. Short-Range Dislocation Glide

The short-range term, $\sigma^{*}$, is the stress needed to move dislocations past short-range obstacles. This is also known as a thermal contribution in which the temperature dependency is strong. It accounts for thermal vibrations to assist dislocations to overcome obstacles. Short range obstacles include all disturbances in the lattice that is small enough, so that thermal vibrations, together with the effective stress, can move a dislocation past that obstacle. These obstacles can be solutes or precipitates. In this work, the solid solution contribution is modeled explicitly and is described in Section 3.4 below.

The Orowan equation describes the relation between the plastic strain rate and the dislocation velocity as

(16) $${\dot{\bar{\varepsilon }}}^{p}=\frac{\rho_{m}b\bar{v}}{M}$$

where $\bar{v}$ is the dislocation velocity, and $\rho_{m}$ is the mobile dislocation density. The dislocation velocity is considered as the time it takes for a dislocation to overcome an obstacle and move to the next. This consist of a waiting time, before the dislocation manage to overcome the obstacle, and a running time, when the dislocation has passed the obstacle and moves to the next. It is assumed that the average velocity only depends on waiting time as the running time is negligible in comparison [9]. The dislocation velocity, $\bar{v}$, is defined as

(17) $$\bar{v}=\lambda v_{a}\exp\left(-\frac{∆G}{kT}\right)$$

where λ is the average obstacle spacing and $v_{a}$ the attempt frequency. Gibbs free energy ∆G is related to the distribution of obstacles and the shape of the barrier the dislocation must overcome. A general form of the activation energy can be written [9]

(18) $$∆G=∆F{\left(1-{\left(\frac{\sigma^{*}}{\tau_{0}G}\right)}^{p}\right)}^{q}$$

where $\tau_{0}G$ is a stress. Now, if the stress increases and $\tau_{0}G$ becomes equal the short-range stress, $\sigma^{*}$, the Gibbs free energy reduces to zero, and then, the dislocations are forced through obstacles. ∆F must be the total free energy required for a dislocation to overcome lattice resistance or obstacles without aid from external stress. The importance of p and q depends on the magnitude of ∆F. If ∆F is large, the values of p and q are unimportant, thus p=q=1 can be used. When ∆F is small, the choice of p and q becomes more critical and should then be fitted to experimental data.

Without aid from external stress, the energy for a dislocation ∆F can be written [9]

(19) $$∆F=∆f_{0}Gb^{3}$$

where $∆f_{0}$ describes the mean obstacle strength. By introducing the constant

(20) $${\dot{\bar{\varepsilon }}}_{ref}=\frac{\rho_{m}\lambda bv_{a}}{M}$$

and now combine Equations (16)–(20), the short-range stress can be written

(21) $$\sigma^{*}=\tau_{0}G{\left(1-{\left(\frac{kT}{∆f_{0}Gb^{3}}\ln\left(\frac{{\dot{\bar{\varepsilon }}}_{ref}}{{\dot{\bar{\varepsilon }}}^{p}}\right)\right)}^{1/q}\right)}^{1/p}$$

Frost and Ashby [9] give ranges for the material parameters $\tau_{0}$ and $∆f_{0}$ for different obstacle strengths. These ranges aid to decide within which boundaries the parameters should be. For an obstacle with medium strength, $∆f_{0}=0.2-1.0,$ and $\tau_{0}=b/l=b\sqrt{\rho_{i}}$. With b taken from Table 3, and $\rho_{i}$ taken as $\rho_{i0}$ from Table 4, an initial value for $\tau_{0}\approx 8\times 10^{-5}$ is a good approximation. The model proposed by Lindgren et al. [14] included dynamic strain aging in Equation (21) but has been excluded in the current work. It is also possible to include Hydrogen Enhanced Localized Plasticity (HELP) in a similar way as shown in [29].

### 3.4. Solid-Solution Strengthening

A strengthening mechanism, where solute atoms are added to the material, causing local non uniformity in the lattice and hindering dislocation motion, is called solid solution strengthening. The model in the current work was proposed by Kou et al. [27]. It is a combination of Labusch approach [30] for binary alloys and the work by Gypen and Deruyttere [31] for multi component alloys. It may be expressed as

(22) $$\sigma_{s}\left(T\right)={\left[\underset{i}{\sum }{\left(Z_{L}G\left(T\right)\right)}^{3/2}{\left(\alpha_{s}\delta_{i}+{\eta^{\prime }}_{i}\left(T\right)\right)}^{2}c_{i}\right]}^{2/3}$$

Thus, the model is a summation of the solutes i, which contributes to a solid solution strengthening. $Z_{L}$ is a material parameter in the range of 1 [32]. A value of $1.3\times 10^{-3}$ has been used for copper [33], whereas the value of $1.8\times 10^{-3}$, which was used for nickel-based alloys [27], is used in the current work. α is a dimensionless constant, $3<\alpha_{s}<16$ for screw dislocations, $\alpha_{s}>16$ for edge dislocations. In this work, $\alpha_{s}$ is chosen to 16 [34]. $\delta_{i}$ is the lattice misfit between the matrix and the solute atom measured by

(23) $$\delta_{i}=a^{-1}\left(\frac{\Delta a}{\Delta c_{i}}\right)=a^{-1}\left(\frac{da}{dc_{i}}\right)$$

where a is the cell parameter, and $c_{i}$ is the atomic concentration. ${\eta^{\prime }}_{i}$ in Equation (22) is calculated through

(24) $${\eta^{\prime }}_{i}\left(T\right)=\frac{\eta_{i}\left(T\right)}{1+\frac{\left|\eta_{i}\left(T\right)\right|}{2}}\;$$

where $\eta_{i}\left(T\right)$ is the temperature-dependent modulus misfit calculated as

(25) $$\eta_{i}\left(T\right)=\frac{G_{0}\left(T\right)-G_{B}\left(T\right)}{G_{B}\left(T\right)}$$

$G_{0}\left(T\right)$ and $G_{B}\left(T\right)$ are the shear modules of the solute and the base metal (Ni), respectively.

The alloying elements with an atomic concentration larger than 1% were included in this contribution. These elements were Cr, Mo, Fe and Ni, both for alloy 625 and alloy 718. The lattice misfits and temperature dependent shear modulus are shown in Table 2.

### 3.5. High Temperature Plasticity, Power Law Breakdown

At elevated temperatures above $0.6T_{m}$ and at stresses larger than $10^{-3}$ of the bulk modulus, the dominating deformation mechanisms change in the material and the flow stress goes from climb controlled to glide controlled. Frost and Ashby [9] propose a relation between stress and plastic strain rate, which they call power law breakdown,

(26) $$\sigma_{PLB}=\frac{G}{\alpha^{\prime }}\mathrm{asinh}{\left(\frac{{\dot{\bar{\varepsilon }}}^{p}kT}{AGbD_{eff}}{\left(\alpha^{\prime }\mathrm{asinh}\left(1\right)\right)}^{n}\right)}^{1/n}$$

where $\alpha^{\prime }$, n and A are material parameters. The effective diffusion coefficient is

(27) $$\;D_{eff}=D_{v}+D_{pipe}$$

$D_{v}$ and $D_{pipe}$ are the lattice and core diffusion, respectively. The core diffusion can be written [9]

(28) $$\;D_{pipe}=\rho_{i}a_{c}D_{c0}e^{-\frac{Q_{c}}{k_{B}T}}$$

It is assumed that the power law breakdown contribution replaces $\sigma^{*}$, $\sigma_{HP}$ and $\sigma_{s}$ at high temperatures. The contributions are then treated as alternatives according to Equation (5).

### 3.6. Solution Procedure

In the procedure of computing the flow stress evolution, the total strain is given in each increment, but the plastic strain increment must be found by iterations. A radial return algorithm can be used for this purpose [35]. This method needs hardening modulus and updated internal state variables. In this work the yield stress model was calibrated and validated with compression tests. The solving procedure for this one-dimensional stress case is described here. The history of the total equivalent strain and temperature is in a discretized form used as input. The equivalent plastic strain, ${\bar{\varepsilon }}^{p}$, and immobile dislocation density, $\rho_{i}$, are internal state variables. The increment of equivalent plastic strain, $\Delta {\bar{\varepsilon }}^{p}$, is calculated in each increment. It is done with a radial return algorithm where $\Delta {\bar{\varepsilon }}^{p}$ is iterated until the yield stress is equal to the effective stress, $\sigma_{eff}$, that is

(29) $$\sigma_{y}-\sigma_{eff}=0$$

where $\sigma_{eff}$ is equal to

(30) $$\sigma_{eff}=\sigma_{y1}+E\left(\Delta \bar{\varepsilon }-\Delta {\bar{\varepsilon }}^{p}\right)$$

where the iteration index of $\Delta {\bar{\varepsilon }}^{p}$ is omitted. $\sigma_{y1}$ is the stress in the beginning of the increment; $\Delta \bar{\varepsilon }$ is the equivalent total strain increment, and E is Young’s modulus. $\Delta {\bar{\varepsilon }}^{p}$ is updated in each iteration as

(31) $${}^{j+1}\Delta {\bar{\varepsilon }}^{p}={}^{j}\Delta {\bar{\varepsilon }}^{p}+\frac{\sigma_{eff}-\sigma_{y}}{E+H^{\prime }}$$

where j gives the iteration number. Equation (31) shows that the yield stress and hardening modulus, $H^{\prime }$, are needed in the radial return method. The hardening modulus is defined as

(32) $$H^{\prime }=\frac{d\sigma_{y}\left(\rho_{i}\right)}{d{\bar{\varepsilon }}^{\;p}}=\frac{d\sigma_{y}\left(\rho_{i}\right)}{d\rho_{i}}\frac{d\rho_{i}}{d{\bar{\varepsilon }}^{\;p}}$$

The yield stress is calculated with the internal state variables, $\rho_{i}$ and $\Delta {\bar{\varepsilon }}^{p}$.

$\rho_{i}$ is updated with a fully implicit approach in a nested loop where $\Delta {\bar{\varepsilon }}^{p}$ and the time step, Δt, are used as inputs. The equation that is solved is in discretized form written as

(33) $$R=\rho_{i}-\rho_{i1}-\left(\frac{M}{b}\frac{1}{\Lambda }\Delta {\bar{\varepsilon }}^{p}-\Omega \rho_{i}\Delta {\bar{\varepsilon }}^{p}-c_{\gamma }D_{v}\frac{Gb^{3}}{k_{B}T}\left(\rho_{i}^{2}-\rho_{i0}^{2}\right)\Delta t\right)=0$$

where the last terms come from Equations (8), (11) and (13). $\rho_{i1}$ is the immobile dislocation density at the beginning of the increment whereas $\rho_{i}$ is at the end of the increment (the iteration index is omitted). The derivative of R with respect to $\rho_{i}$ is needed in the iterative procedure and is calculated as

(34) $$\frac{dR}{d\rho_{i}}=1+\Omega \Delta {\bar{\varepsilon }}^{p}-2c_{\gamma }D_{v}\frac{Gb^{3}}{k_{B}T}\rho_{i}\Delta t$$

Now $\rho_{i}$ is updated in each iteration as

(35) $${}^{j+1}\rho_{i}={}^{j}\rho_{i}-R{\left(\frac{dR}{d\rho_{i}}\right)}^{-1}$$

where j gives the iteration number.

## 4. Calibration and Validation

In this section, the calibration and validation results of the material model will be presented. The stress and strain from the experiment is originally negative in sign since it is uniaxial compression tests. However, for the ease of reading and later implementation in finite element software, the sign is changed to positive for both stress and strain. Figures of the measured and computed flow stress curves and tables for the obtained material parameters will be shown. Some of the material parameters were obtained from literature. Parameters belonging to the solid solution strengthening model are shown in Table 2 and other parameters are shown in Table 3.

### 4.1. Calibration

**Figure 2:**
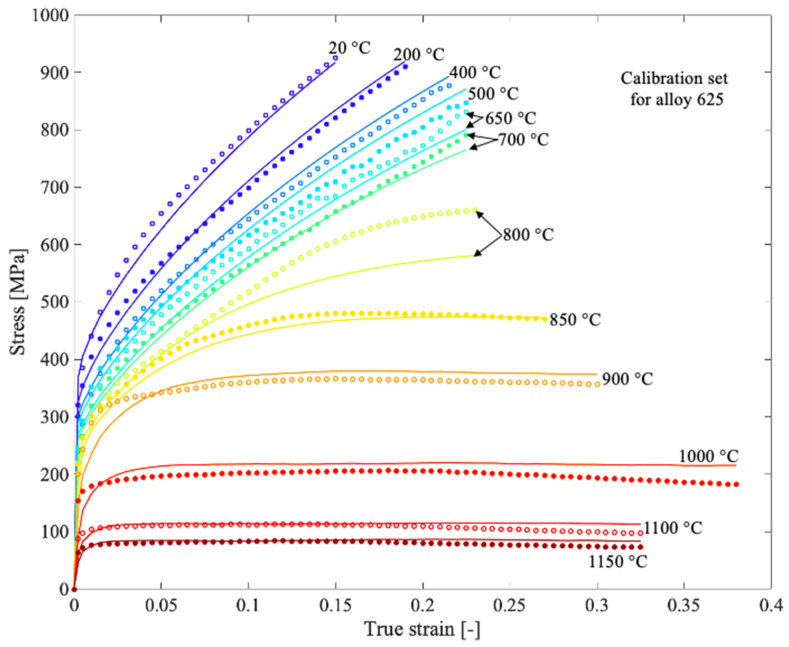
Measured flow stress curves (discrete points) for alloy 625 and corresponding computed result (solid lines) from the material model after calibration. The tests were performed with a nominal strain rate of 0.01 $s^{-1}$.

**Figure 3:**
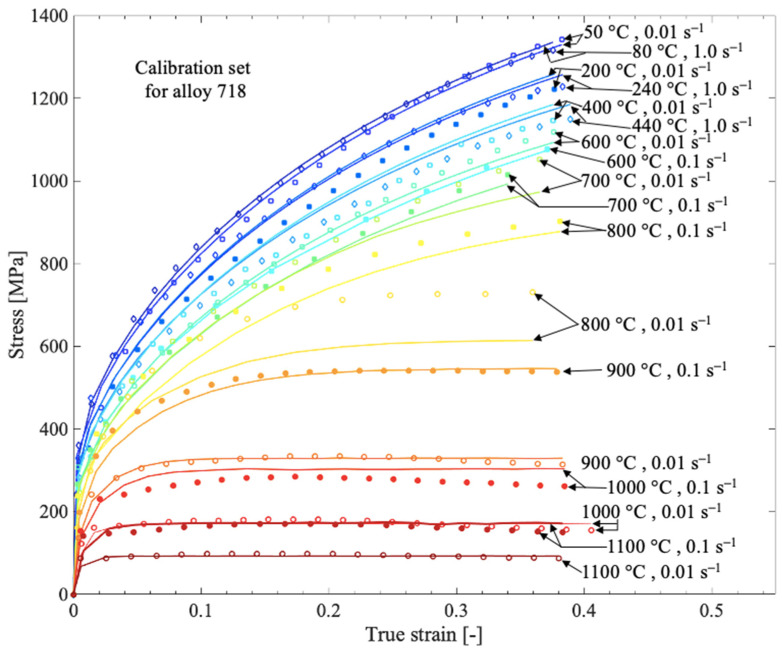
Measured flow stress curves (discrete points) for alloy 718 and corresponding computed result (solid lines) from the material model after calibration.

Since the composition of the alloys in this work is similar, the same values were used for both alloy 625 and alloy 718, although differences may be found. For alloy 625, all measured compression tests used a nominal strain rate of 0.01 $s^{-1}$, whereas for alloy 718, strain rates with a range of 0.01–1 $s^{-1}$ were used (the actual strain rate in the tests differs somewhat from the nominal strain rate and is accounted for in the model). The calibrated results are shown in Figure 2 and Figure 3 for alloy 625 and alloy 718, respectively, and the calibrated parameters are shown in Table 4. The calibration was performed with an error minimization procedure using a toolbox developed in Matlab. Before the optimization procedure started, the stress–strain curves were smoothed.

### 4.2. Validation

**Figure 4:**
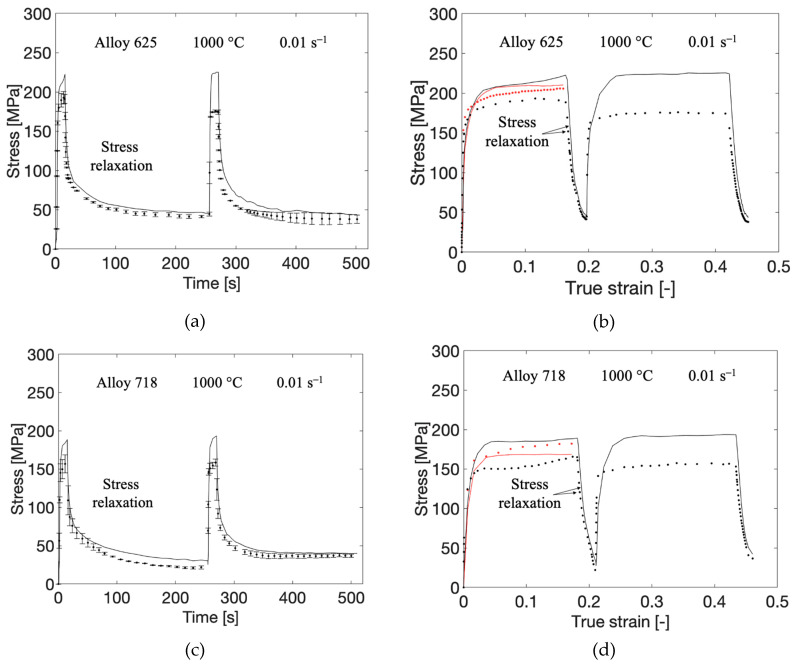
Measured (discrete points) and computed (solid lines) stress relaxation tests for alloy 625, shown in (**a**,**b**), and alloy 718, shown in (**c**,**d**). (**a**,**c**) include errorbars for the measured curves. In (**b**,**d**), the ordinary compression tests from the calibration sets are included. The difference in stress level of the modeled curves in the first sequence is because of variations in strain rate in the experiments.

**Figure 5:**
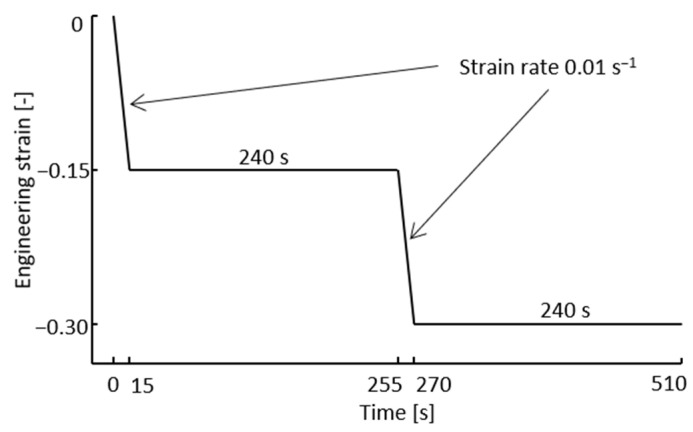
Loading path for the stress relaxation tests.

**Figure 6:**
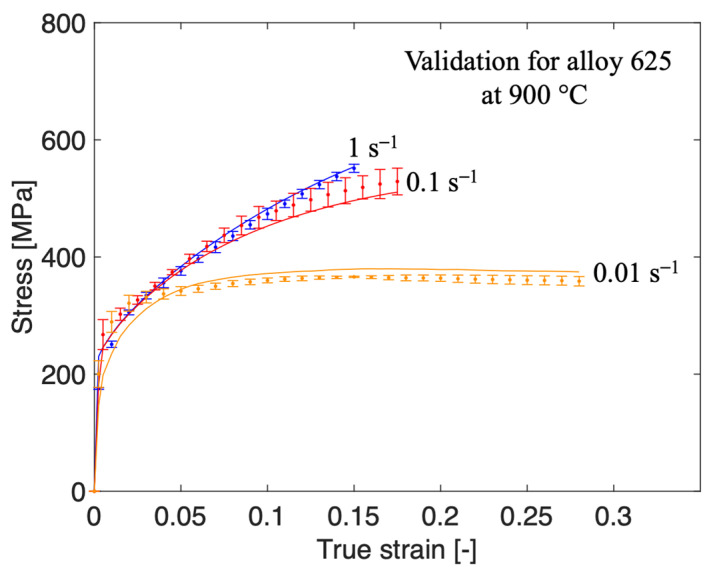
Measured (discrete points with errorbars) and computed (solid lines) flow stress curves at 900°C at different strain rates. The curves for 0.01 $s^{-1}$ belong to the calibration set whereas the other curves (for 0.1 $s^{-1}$ and 1 $s^{-1}$ ) are predicted by the material model.

The model with the optimized parameters was subsequently validated by comparing with stress relaxation tests. The results are presented in Figure 4a–d. The stress relaxation tests for both alloys were performed in accordance with the uniaxial compression tests detailed in chapter 2, apart from the loading path. The loading path for the stress relaxation tests can be seen in Figure 5. The tests are performed at a temperature of 1000 °C and are repeated two times with the difference shown in the error bars. In the first step, an engineering strain of 15% is applied at a strain rate of 0.01 $s^{-1}$. The strain is then kept constant for 240 s. During this step, the induced stress is expected to relax to some extent. The second loading is then applied with the same strain rate and the same relative increase in strain, so a total of 30% engineering strain is achieved. The final part is the holding at constant strain for another 240 s. The red curves in Figure 4b,d represent the corresponding compression test and simulation result from Figure 2 and Figure 3, respectively. The stress levels of both the experimental and modeled curves differ slightly while they are comparable (in the first loading sequence of the tests). This is due to different effective strain rates as the load path is somewhat different. The root mean square error (RMS) between the measured and modeled curve was also calculated to 31.6 and 32.0 for the relaxation of alloy 625 and alloy 718, respectively. The strain rate sensitivity of the model was also validated with additional compression tests of alloy 625 at 900 °C and nominal strain rates of 0.1 $s^{-1}$ and 1 $s^{-1}$, shown in Figure 6. The RMS values between the modeled and measured curves were 13.9 for the test at 1 $s^{-1}$ and 19.6 for the test at 0.1 $s^{-1}$, respectively.

**Figure 7:**
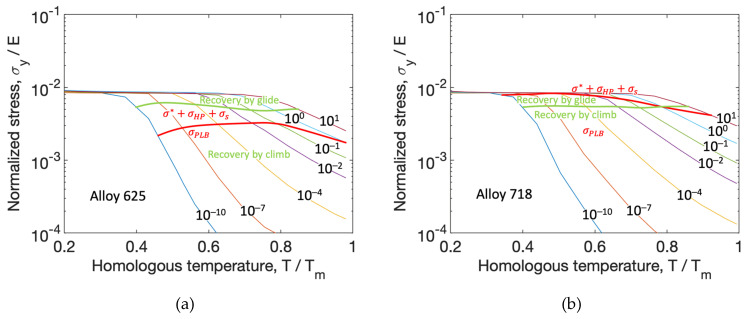
Deformation mechanism maps for alloy 625 (**a**) and alloy 718 (**b**). The x-axis shows the temperature that is divided with the melting temperature (in kelvin), and the y-axis shows the flow stress at steady state that is divided with Young’s modulus.

Deformation mechanisms maps were constructed and are shown in Figure 7. Inspiration for the creation of the maps has been taken from Frost and Ashby [9]. The curves in the maps represent different strain rates (from $10^{-10}$ to $10^{1}$ $s^{-1}$). The green boundary separates the map in two regions. Above the boundary, recovery by glide (Equation (11)) dominates, whereas below the boundary, recovery by climb (Equation (13)) dominates. The red boundary also separates the map into two regions and shows if $\sigma^{*}+\sigma_{HP}+\sigma_{s}$ or $\sigma_{PLB}$ is active in the model; see Equation (5). They also show regions where different mechanisms are dominating.

## 5. Discussion

A physically based material model has been optimized from RT to 1150 °C for alloy 625 as well as for alloy 718 in its solution annealed condition. Both alloys are superalloys and have a similar composition. All known or assumed parameters are the same for both materials. An optimization procedure was used to determine a total of 15 parameters. The strain rate dependency in the recovery by glide (Equation (12)) was found to be negligible, so in practice, only 12 parameters needed calibration. For alloy 718, two strain rates were used: 0.01 and 0.1 $s^{-1}$ from 600–1100 °C and 1 and 0.01 $s^{-1}$ from RT, 200 and 400 °C, during the optimization. For alloy 625, on the other hand, only one strain rate was used. The optimized curves are in good agreement with the measured curves for both alloys except at a temperature around 800 °C. At this temperature, the model gave a significant lower flow stress, compared to the measured flow stress, for both alloys. Although only one strain rate was used for alloy 625 in the optimization procedure, the predictability of the model is very good for other strain rates as shown in Figure 6. This shows that it might not be necessary to calibrate the model using a full set of strain rates for all temperatures.

One major advantage over a purely empirical model is that a mechanism-based flow stress model can account for material recovery. This is because of the recovery in dislocation density is accounted for in the model. Note that the model can be implemented in a finite element code in the same way as, for example, Johnson–Cook representing the evolution of the yield surface of, e.g., von Mises. The predictability of the material recovery is demonstrated in the relaxation test shown in Figure 4. It should be noted that the relaxation test is only repeated two times, but as seen on the error bars in the plot, there is no large variation between the tests. The measured curve for alloy 625 shows that the flow stress in the first cycle does not reach as high level as in the second cycle (see Figure 4b). The same is true for alloy 718 but to a smaller extent. The model does not capture this behavior and gives the same flow stress level in both sequences. One reason for this behavior could be that the recrystallization occurs during the relaxation procedure. The evolution equation describing the increase in dislocations, as well as the Hall–Petch contribution, includes the grain size. However, this behavior is not possible to predict since there is no grain growth model coupled to the flow stress model. This hypothesis remains to be verified by experiments first though.

Using the model, deformation mechanisms maps have been generated. Both alloys have the change in recovery mechanism activated at the same temperature and normalized stress level, but a different stress level for when PLB is activated is seen between the two alloys. For alloy 625, the mechanism is active below recovery by glide, whereas for alloy 718 it is active above recovery by glide. This shows that the mechanism for high-temperature plasticity for alloy 625 is active at higher temperatures than for alloy 718. Based on this, it is indicated that alloy 625 is more resistant to creep than alloy 718.

For elevated temperatures, the expressions for thermally active mechanisms are replaced with one expression, the power-law breakdown. It is an empirical expression and starts to dominate for temperatures above approximately 800 °C, whereas the flow stress is modeled using physically based expressions otherwise. For the sets of flow stress curves in the current work, this empirical contribution worked well. However, physically based contributions often have more extensive validity ranges compared to empirically based. Therefore, the flow stress model can be improved if the physical mechanisms that result in this power-law behavior are implemented. A model for dynamic recrystallization (DRX) [37], or a model for globularization [13], which is a similar process as DRX, may replace the power-law breakdown model since it is known that DRX occurs both for alloy 625 [38] as well as for alloy 718 [39].

Alloy 625 has about the same overall flow stress as alloy 718 at low temperatures (20–200 °C), lower at intermediate temperatures (400–700 °C), and higher for temperatures close to melting temperature (800–1100 °C). Dynamic strain ageing and solid solution strengthening may be the reasons for this behavior. For alloy 718, dynamic strain ageing is attributed to diffusion of Nb atoms, but for alloy 625, it is attributed to Mo atoms in the lattice [40]. At even higher temperatures (800–1100 °C), it is suggested to be the solid solution strengthening of alloy 625 that gives the higher flow stress owing to the higher total content of the element, Nb and Mo.

## 6. Conclusions

The mechanism-based material model has been calibrated and validated for two commonly used superalloys, alloy 625 and alloy 718. A model for solid solution strengthening and contribution for high temperature plasticity were implemented in the material model. The model naturally accounts for relaxation and can be implemented in the same fashion as an empirical model into a finite element program, allowing the yield surface to depend on the strain, strain rate and temperature. The following conclusions can be drawn from the current work:

- The model has been calibrated versus compression tests in strain rates of 0.01–1 $s^{-1}$ and a temperature range from room temperature (RT) up to temperatures close to the melting temperature (1150 °C).
- The material parameters were calibrated to almost the same values for both alloys. This is physically sound since the materials have similar composition, and it also shows the generality and robustness of the model.
- Although only one strain rate was used in the calibration for alloy 625, the material model could predict the flow stress for different strain rates at 900 °C.
- The model naturally accounts for material recovery and captured the stress relaxation in validation tests at 1000 °C.
- The deformation mechanism maps show that both alloys have the change in recovery mechanism activated at the same temperature and normalized stress level. The transition to the power-law breakdown mechanism ($\sigma_{PLB}$) occurs at higher temperatures for alloy 625.

## Figures and Tables

**Table 1:** Chemical composition (wt %) for alloy 625 and alloy 718.

**Material**	**Ni**	**Cr**	**Mo**	**Fe**	**Nb**	**Ti**	**Al**	**Si**
Alloy 625	Bal	22.31	9.03	4.37	3.58	0.22	0.19	0.16
Alloy 718	53.5	18.4	3.05	Bal	5.00	0.94	0.6	0.08
**Material**	**Co**	**Mn**	**Cu**	**Ta**	**C**	**P**	**S**	
Alloy 625	0.071	0.063	0.033	0.020	0.015	0.005	0.001	
Alloy 718	0.17	0.11	0.13	-	0.03	0.010	0.0004	

**Table 2:** Lattice misfits, δ, and temperature dependent shear modulus (GPa) for alloy 625 and alloy 718.

Element	Ni	Cr	Mo	Fe	Nb
	**Lattice Misfits** [27]				
		0.0134	0.0400	0.00424	0.0671
**Temperature (°C)**	**Shear Modulus (GPa)** [9]				
20	79.1	126	134	81.3	44.3
100	76.8	124	133	78.0	44.3
200	73.8	121	131	73.9	44.3
300	70.9	118	129	69.9	44.3
400	68.0	115	127	65.8	44.3
500	65.1	112	125	61.7	44.3
600	62.1	109	123	57.7	44.3
700	59.2	106	121	53.6	44.3
800	56.3	104	119	49.5	44.3
900	53.4	101	117	45.4	44.3
1000	50.4	97.7	115	41.4	44.3
1250	43.1	90.4	110	31.2	44.3

**Table 3:** Known or assumed parameters used in the flow stress model both for alloy 625 and alloy 718.

Parameter	Unit	Comment	Values	
$k_{B}$	J/K	Boltzmann’s constant	$1.38\times 10^{-23}$	
M	−	Taylor factor for fcc unit cell	3.06	[9]
b	m	Burgers vector	$2.5\times 10^{-10}$	[9]
${\dot{\bar{\varepsilon }}}_{ref}$	$s^{-1}$	Reference strain rate	$1.0\times 10^{6}$	[9]
$D_{v0}$	$m^{2}/s$	Lattice self-diffusion coefficient	$1.6\times 10^{-4}$	[9]
$Q_{v}$	kJ/mol	Activation energy for lattice diffusion	285	[9]
$a_{c}D_{c0}$	$m^{2}/s$	Core diffusion coefficient	$1.0\times 10^{-25}$	[9]
$Q_{c}$	kJ/mol	Activation energy for core diffusion	170	[9]
g	μm	Grain size	20	
a	m	Cell parameter	$3.6\times 10^{-10}$	[36]

**Table 4:** Parameter values obtained from the optimization procedure.

Parameter	Unit	Value, Alloy 625	Value, Alloy 718	Equation
α	-	0.524	1.0	(6)
$\rho_{i0}$	$m/m^{3}$	$1\times 10^{11}$	$1\times 10^{11}$	(6),(10),(11) ${}^{1}$
$∆f_{0}$	-	0.25	0.25	(21)
$\tau_{0}$	-	0.0089	0.028	(21)
p	-	0.21	0.12	(21)
q	-	1.65	1.65	(21)
$K_{c}$	-	62.5	154	(10)
$\Omega_{0}$	-	4.7	4.5	(12)
$\;x_{d}^{\max}$	-	$-{\;}^{2}$	$-{\;}^{2}$	See Lindgren et al. [14]
$\Omega_{r0}$	-	0	0	(12)
Q	kJ/mol	$-{\;}^{2}$	$-{\;}^{2}$	(12)
$c_{\gamma }$	-	2.5	6.3	(13)
A	-	3.5	4.1	(26)
n	-	3.2	2.9	(26)
$\alpha^{\prime }$	-	500	600	(26)

${}_{}^{1}\rho_{i0}$ is the initial value of the internal state variable $\rho_{i}$, ${}_{}^{2}$ Have no influence in the model when $\Omega_{r0}$ was calibrated to be zero.
